# Acute Kidney Injury Complicated Epstein-Barr Virus Infection in Infancy

**DOI:** 10.1155/2015/848959

**Published:** 2015-05-04

**Authors:** Gamze Ozgurhan, Mustafa Ozcetin, Aysel Vehapoglu, Zeynep Karakaya, Fatih Aygun

**Affiliations:** ^1^Department of Pediatrics, Suleymaniye Maternity and Children's Training and Research Hospital, Istanbul, Turkey; ^2^Department of Pediatrics, Bezmialem Vakif University Medical School, Istanbul, Turkey; ^3^Department of Pediatric Intensive Care, Istanbul University Cerrahpasa Medical School, Istanbul, Turkey

## Abstract

Infectious mononucleosis is an acute lymphoproliferative disorder caused by the Epstein-Barr virus (EBV) and seen most commonly in children and young adults. Clinical presentation of the disease is characterized by fever, tonsillopharyngitis, lymphadenopathy, and hepatosplenomegaly, whereas serological findings of this benign disorder include positive heterophilic antibody formation (transient increase in heterophilic antibodies) and prominence of hematological lymphocytosis of more than 10% of atypical lymphocytes. An EBV infection is usually asymptomatic in childhood, but acute kidney injury can be a rare complication during its course. Most cases recover from the disease completely. Early recognition of EBV infection and estimation of its complication are important for its prognosis. In light of previous literature, we discuss the case evaluated as an EBV infection complicated by acute kidney injury in early childhood and results of tubulointerstitial nephritis shown on a renal biopsy that was later diagnosed as an EBV infection by serological examination.

## 1. Introduction

The Epstein-Barr virus (EBV) affects almost all the systems of the body and therefore has a broad spectrum of clinical outcomes. It was discovered by Epstein, Achong, and Barr on microscopic examination of cell cultures obtained from Burkitt lymphoma 50 years ago [1]. In 1968, EBV was demonstrated as a causative agent for heterophile-positive infectious mononucleosis. In the 1970s, it was found in certain tissues of nasopharyngeal carcinoma patients. Finally in the 1980s, a correlation between EBV and oral hairy leukoplakia and non-Hodgkin's lymphoma was proven in patients with AIDS [2]. The most common clinical feature seen in adults and adolescents with infectious mononucleosis is a triad of fever, sore throat, and lymphadenopathy. Serological tests showing positive heterophilic antibodies and peripheral lymphocytosis with atypical lymphocytes have been determined [3]. In infants and young children, nonspecific and subclinical symptoms are usually observed [4]. In some cases, a primary EBV infection remains silent and clinically atypical until this period. Clinical signs related to almost all affected organs of the body appear as an atypical type of infection with EBV. Diagnosis is made by specific serological tests [5]. Healthy cases generally recover from primary EBV infection completely. However, it can be complicated by renal, cardiac, pulmonary, neurological, and hematological problems [6]. Acute kidney injury related to acute EBV infection has been rarely demonstrated in the literature [7, 8]. In the light of the literature, we discuss here a case of acute kidney injury related to EBV infection in a 13-month-old male patient.

## 2. Case

A previously healthy 13-month-old male patient was admitted to the hospital presenting four days of fever and rash. Before admission, the patient had used amoxicillin and clavulanic acid treatment in an appropriate dose for an upper respiratory tract infection, but the fever did not subside and his body temperature increased to 40°C with shivering. He had no complaints other than fatigue. His past history and family history were unremarkable.

On physical examination, he appeared fatigued with body weight of 9750 g (25 p); height of 76 cm (25–50 p); axillary temperature of 39.8°C; blood pressure of 100/60 mmHg; heart rate of 130/min; and respiratory rate within normal range. Lymphadenopathy and organomegaly were not present. There were no respiratory, cardiovascular, gastrointestinal, or neurological signs, but fever, mild hyperemia of pharynx, maculopapular rash that blanches under pressure, and some petechial rashes on lower limbs.

On total blood count, Hb was 10.4 g/dL and WBC was 17,060/mm^3^ (36.2% neutrophil, 48.4% lymphocytes, and 11% atypical lymphocytes), and PLT count was found normal (309,000/mm^3^). Liver function and renal function tests and serum electrolytes were found normal. Due to the presence of persistent fever and rash, viral serological tests for isolation of etiological agent (TORCH, parvovirus, EBV VCA IgM, and EBV VCA IgG), monospot tests, rose bengal tests, tube agglutination tests for* Brucella*, urine, and stool analysis, and blood cultures were investigated. On examination, direct stool smear was normal. Urine analysis showed no significant results other than (+) proteinuria, 7 leucocytes, and 2 erythrocytes. For differential diagnosis and exclusion of atypical Kawasaki disease, an echocardiogram was performed and found normal. Monospot tests, rose bengal tests, and tube agglutination tests for* Brucella* were negative.

On the fourth day of admission, due to low urine output and bilateral orbital edema, laboratory tests were repeated with results as follows: WBC: 16,600/mm^3^, hemoglobin: 10 g/dL, and PLT: 173,000/mm^3^. Serum electrolytes include sodium: 135 mEq/L; potassium: 7.09 mEq/L, chlorine: 107 mEq/L; bicarbonate: 11.5 mEq/L; urea: 181 mg/dL; creatinine: 4.1 mg/dL; calcium: 7.3 mg/dL; albumin: 2.95 g/dL; uric acid: 3.11 mg/dL; and mild elevation of transaminases (aspartate aminotransferase: 170 U/L and alanine aminotransferase: 79 U/L). Bilirubin and alkaline phosphatase were found to be normal. All types of cultures sent were found sterile.

An abdominal ultrasound showed the right and left kidney long axes to be 82 mm and 83 mm, respectively, and increased in length (>95th percentile for age). Both kidneys had grades 1-2 parenchymal hyperechogenicity. Sonography showed free collections at various sites, including perihepatic, perisplenic, and lower quadrant of the abdomen with the largest site measuring 8 mm.

The first attempt for treatment was fluid restriction (urine output + insensible losses), sodium bicarbonate (1 mEq/kg), and calcium gluconate (1 mL/kg). Later, the patient developed oligoanuria, features of acute kidney injury, and metabolic acidosis; therefore, hemodialysis treatment in the pediatric intensive care unit was applied. Serological testing for EBV VCA IgM and EBV VCA IgG performed at admission was positive. Hence EBV nuclear antigen (EBNA) was checked and found to be negative. No evidence for acute infection was determined in other serological tests. ASO was found negative. C3 complement level was normal. Early examination of renal biopsy material showed intense and mixed tubulointerstitial inflammatory infiltration rich with T cells and histiocytes. Immunofluorescence studies for IgG, IgA, IgM, C3, fibrinogen, C19, kappa, and lambda were negative. Immunohistochemical studies for CMV, EBV, HSV I/II, and parvovirus were also found negative.

Due to the presence of peripheral atypical lymphocytosis and positive serological tests for EBV, the case was evaluated as acute kidney injury related to interstitial nephritis secondary to atypical EBV infection in early childhood. The patient needed sequential hemodialysis due to acute kidney injury and metabolic acidosis and complete recovery of renal functions (urea: 29 mg/dL; creatinine: 0.4 mg/dL; sodium: 138 mEq/L; potassium: 4.6 mEq/L) occurred in about one month.

## 3. Discussion

The Epstein-Barr virus usually appears as infectious mononucleosis in adolescents and adults, whereas it has asymptomatic and nonspecific symptoms in infants and children [2–4]. We present a case where a 13-month-old infant, who had been under treatment for acute infection, developed acute kidney injury and was duly followed up in pediatric intensive care unit.

Infectious mononucleosis, when it has significant clinical features, presents as a triad of fever, lymphadenopathy, and pharyngitis in half of the patients. Rarely being atypical, it can be complicated with pneumonia, shock, blood dyscrasias, fulminant hepatitis, encephalitis, carditis, arthritis, uveitis, and pancreatitis [1]. These rare features make the diagnosis of infectious mononucleosis and its differential diagnosis for Kawasaki disease difficult, especially in early phases of the infection. So far, one of the rarest complications caused by infectious mononucleosis is acute kidney injury. In our case, atypical features of infectious mononucleosis with development of acute kidney injury requiring sudden hemodialysis and diagnosis as EBV infection using serological tests are represented. Typical laboratory findings of infectious mononucleosis are atypical lymphocytosis (>10%) with absolute lymphocytosis, positive heterophilic antibodies, and mild-to-moderate elevation of serum aminotransferases. The presented case demonstrated 17060 leucocytes and 11% atypical lymphocyte count. Heterophilic antibody response is not generated well in children under 10, a well-known reaction also in line with the response in our case.

Serological profiles of EBV antibodies are quite characteristic and necessary for diagnosis of atypical infections [9]. In our case, there were no particular signs of EBV infection and diagnosis was based on serological examination and elevation of both EBV VCA IgM and EBV VCA IgG. It is expected that EBV infection coupled with amoxicillin use can cause a rash, which was also the case with our patient.

In EBV infections, a true renal parenchymal involvement is rarely found, although abnormalities in urine sediment can be seen in 5–15% of cases [10, 11]. Wechsler et al. [12] reported that 17 out of 556 cases presented abnormalities in urine analysis such as microscopic hematuria and proteinuria without renal parenchymal involvement. Lee and Kjellstrand described 14% of proteinuria and 11% of hematuria in 128 EBV-infected cases [13]. In another study, where a series of cases of infectious mononucleosis without clinical findings related to renal illness are studied, swelling in glomerular cells and focal interstitial mononuclear infiltration in renal biopsy are found in 12 out of 13 patients [14].

Rhabdomyolysis and hepatic failure are the leading causes of EBV-related acute kidney injury [7]. In some cases, isolated tubulointerstitial nephritis, mesangial proliferation, and tubular necrosis result in kidney injury as well [7, 8, 13]. Mayer et al. examined EBV-associated kidney injury cases and found 3 with rhabdomyolysis, 2 with glomerulonephritis, 1 with minimal change disease, 1 with hemolytic uremic syndrome, and 1 with interstitial nephritis relevant acute kidney injury out of 13 cases with ages ranging from 4 to 18 years. In the majority of these cases, kidney injury patients had recovered completely within one to two weeks, whereas only one patient required dialysis. In case of the patient with interstitial nephritis, the patient required renal transplantation despite treatment with prednisolone [7]. In 14 patients with glomerular abnormalities with infectious mononucleosis, Ramelli et al. found the glomerular pathology being quite diverse, ranging from minimal change to focal sclerosis, and proliferative or sclerosing glomerulonephritis [15].

Tubulointerstitial nephritis is an unusual cause of acute kidney injury in pediatric patients. Greising et al. reported 7% of child patients having tubulointerstitial nephritis among all who went on renal biopsy. In this series, FSGS and interstitial nephritis were detected during renal biopsy in the case of a 15-year-old patient with acute mononucleosis [16]. Ellis et al. linked only two cases out of 13 TIN patients between 5 and 16 years old with nonspecific viral infections [17]. In our case, acute kidney injury is found to emerge due to interstitial nephritis. Although it appeared in early childhood, the patient required hemodialysis as acute kidney injury and metabolic acidosis developed, and his renal functions recovered completely within one month.

In summary, it should be kept in mind that, in early childhood, unexpected and abrupt emergence of acute kidney injury features in cases with fever can be caused by EBV as an etiological agent; therefore, serological tests should be performed. Renal biopsy done in early periods of the disease can further clarify the diagnosis by determining classical tubular cell infiltration.
